# Efficient direct shoot organogenesis and genetic stability in micropropagated sacha inchi (*Plukenetia volubilis* L.)

**DOI:** 10.1186/s13104-020-05257-1

**Published:** 2020-09-03

**Authors:** Catalina Restrepo-Osorio, Alejandro Gil-Correal, Lina Chamorro-Gutiérrez, Viviana Ramírez-Ríos, Javier C. Álvarez, Diego Villanueva-Mejía

**Affiliations:** Department of Biological Sciences, CIBIOP Research Group, Universidad EAFIT, Medellín, Colombia

**Keywords:** Adventitious shoots, Rooting, Acclimatization, Oilseed, Tissue culture

## Abstract

**Objective:**

It is necessary to improve biotech platforms based on in vitro cell tissue culture to support sacha inchi (*Plukenetia volubilis* L.) research programs and draw on the nutritional value of the high polyunsaturated fatty acid content of its oilseed. Here, we developed a rapid and efficient method for induction and direct in vitro shoot development for this species.

**Results:**

Shoots were generated from hypocotyl explants. The highest organogenic response was obtained in woody plant medium supplemented with 1 mg/L thidiazuron and 0.5 mg/L zeatin supplemented with L-glutamine, adenine hemisulfate, and L-arginine. Shoots obtained using this medium were transferred and subcultivated with different concentrations of indole-3-butyric acid and 1-naphthylacetic acid for rooting. For the first time, a histological analysis was performed supporting direct organogenic development in this species. The plantlets obtained were transferred ex vitro with a survival percentage of 80%. The genetic stability of the plants recovered was confirmed by randomly amplified polymorphic DNA analysis. All results indicate that it would be possible to stimulate direct shoot formation from hypocotyls to support the sustainable use of this species.

## Introduction

Sacha inchi (*Plukenetia volubilis* L.) is a perennial, woody, oleaginous climbing plant belonging to the Euphorbiaceae family [1] that is native to the rainforest of South America. This crop grows in disturbed wet lowland forest at elevations of up to 900 m in the Lesser Antilles and Surinam, and along the northern and western edge of the Amazon basin in Venezuela (Amazonas), Colombia, Ecuador, Peru, Bolivia, and Brazil (western Amazonas) [2]. Its oilseed has excellent nutritional value due to its high content of polyunsaturated fatty acids, including ⍺-linolenic (C18:3, ω-3) and linoleic (C18:2, ω-6) acids [3], which are essential components of the human diet and help to prevent coronary heart disease and hypertension [4].

Several scientific reports have focused on regeneration from hypocotyls and epicotyls in the Euphorbiaceae family [5, 6], despite high recalcitrance. Regarding sacha inchi specifically, a few studies have reported the use of in vitro propagation to evaluate the auxin:cytokinin ratio in apical meristems [7] and different segments of the epicotyl and hypocotyl [8–10], with highest shooting but low process efficiency with 0.5 and 1 mg/L 6-benzylaminopurine and 0.1 mg/L and 0.25 mg/L 1-naphthylacetic acid (NAA). Therefore, it is imperative that scientific knowledge regarding in vitro establishment and effective propagation methods for this promising species is expanded as demand for its oilseed has recently increased worldwide. Such a science-based approach could support research programs on topics such as metabolite production by cell suspension [11] and genetic improvement using modern techniques that seek to overcome the issues of biotic and abiotic stress and enhance crop productivity and fatty acid content [12]. Here, a de novo organogenesis process was induced in sacha inchi and the response of hypocotyl segments under in vitro conditions was evaluated. Furthermore, histological analysis of direct shoot organogenesis was reported for the first time in this species, meaning that this scientific report represents one of the first significant advances in the micropropagation of sacha inchi.

## Main text

### Materials and methods

#### Plant material and culture initiation

Mature sacha inchi fruits were collected at Santa Rosa de Osos (N6°32′42.5′′; W075°13′49.6′′), Antioquia, Colombia, under a permit issued by the National Authority for Environmental Licenses (ANLA; resolution 1312 of 2015). Once transferred to the Plant Biotechnology Laboratory at Universidad EAFIT, the seed coats were removed, then the seeds were washed with soapy water, hydrated in a humid chamber for 12 h, disinfected using 2 ml/L Timorex Gold® 3 h, and incubated in 50 mg/L vancomycin and 250 mg/L cefotaxime for 4 h. Afterward, seeds were rinsed with 70% ethanol for 1 min and 2% v/v sodium hypochlorite for 10 min. All steps included three sterile distilled water rinses. Next, seeds were cultured in woody plant medium (WPM) [13] supplemented with 30 g/L sucrose and 2 g/L activated charcoal and solidified with 1.8 g/L phytagel (pH 5.7). Seeds were maintained in total darkness for 30 d at 25 ± 1 °C under a 12 h photoperiod with fluorescent white light lamps (TL5 14 W; Sylvania™), under 32.43 µmol/m^2^s luminous intensity. After 30 d, germination rates were calculated.

#### Induction of multiple shoots and regeneration of plantlets

Once 2-week-old in vitro plantlets were obtained, hypocotyl sections were excised and placed in Petri dishes containing WPM supplemented with 0.5 mg/L CuSO_4_ or 50 mg/L L-glutamine, adenine hemisulfate, and L-arginine (G + A + A) (Table 1). Proximal and distal segments were taken from the hypocotyls to determine the effect of the cotyledonary node. Cytokinins [thidiazuron (TDZ), zeatin, 6-γ, γ-dimethylallylamino purine, 6-benzylaminopurine, and kinetin] and auxins [indole-3-butyric acid (IBA) and NAA] at concentrations of 0.5 and 1.0 mg/L were assessed. Control plants were grown without growth regulators. Three assays were performed at different times. Each experiment was performed on 15 hypocotyl sections with three replications per treatment. After 45 d, the callus percentage, the average number of shoots per explant, and the number of developed shoots were determined (Table 1).

**Table 1:** Effect of hypocotyl section (proximal or distal), supplements, and growth regulators on the total number of shoots, average shoots per explant, percentage of callus, and percentage of elongated shoots of > 2.5 cm of *Plukenetia volubilis* hypocotyls after 45 d of cultivation

Components	Hormonal treatments	Average of shoots per explant		Percentage of callus		Percentage of elongated shoots (> 2.5 cm)	
		Proximal	Distal	Proximal	Distal	Proximal	Distal
CuSO_4_	TDZ (1 mg/L)	3.04^ab^	1.91^bc^	68.89^a^	72.78^a^	46.74^a^	29.63^a^
	TDZ (1 mg/L) + 2ip (0.5 mg/L)	3.11^ab^	1.73^ab^	73.33^a^	75.0^a^	45.81^a^	25.5^a^
	TDZ (1 mg/L) + Zeatin (0.5 mg/L)	4.62^d^	2.31^c^	72.22^a^	74.44^a^	51.74^ab^	37.96^a^
	TDZ (1 mg/L) + BAP (0.5 mg/L)	3.93^cd^	1.53^ab^	76.11^a^	77.78^a^	48.54^a^	26.67^a^
	TDZ (1 mg/L) + Kinetin (0.5 mg/L)	3.51^bc^	1.62^ab^	75.56^a^	78.33^a^	33.19^a^	28.52^a^
	TDZ (1 mg/L) + IBA (0.5 mg/L)	3.42^abc^	1.47^ab^	75^a^	75.56^a^	41.33^a^	26.67^a^
	TDZ (1 mg/L) + NAA (0.5 mg/L)	3.36^abc^	1.8^b^	76.11^a^	79.44^a^	40.70^a^	27.04^a^
	Control	2.71^a^	1.27 ^a^	13.33^b^	11.11^b^	69.26^b^	68.52^b^
G + A + A	TDZ (1 mg/L)	3.33^abc^	1.76^bc^	70^b^	73.33^a^	41.04^ab^	30.37^a^
	TDZ (1 mg/L) + 2ip (0.5 mg/L)	3.29^ab^	1.8^bc^	73.33^bc^	72.33^a^	44.56^ab^	27.41^a^
	TDZ (1 mg/L) + Zeatin (0.5 mg/L)	5.11^e^	2.2^c^	73.89^bc^	75.0^a^	53.34^b^	36.67^a^
	TDZ (1 mg/L) + BAP (0.5 mg/L)	4.87^de^	1.62^b^	76.11^bc^	77.78^a^	45.69^ab^	29.63^a^
	TDZ (1 mg/L) + Kinetin (0.5 mg/L)	4.11^cd^	1.51^ab^	76.07^bc^	76.67^a^	31.51^a^	27.41^a^
	TDZ (1 mg/L) + IBA (0.5 mg/L)	3.71^abc^	1.6^b^	78.33^bc^	76.11^a^	45.70^ab^	29.63^a^
	TDZ (1 mg/L) + NAA (0.5 mg/L)	3.93 ^bc^	1.73 ^bc^	78.89 ^c^	80.0^a^	38.56 ^ab^	28.15 ^a^
	Control	3.0^a^	1.09^a^	14.44^a^	11.11^b^	77.26^c^	65.93^b^

Different letters indicate significant differences (p ≤ 0.05)

#### Histological observation

Histological analysis was performed during the shoot formation process. Hypocotyl sections of 1 cm in length with meristematic growth zones were set in a mixture of formaldehyde, ethanol, and acetic acid for 24–48 h at 6 °C, and dehydrated in a graduated alcohol series (70%, 80%, 90%, 95%, and 100%). Two lightning steps in xylenes were carried out (4 h each step) and the hypocotyl sections were submerged in Paraplast Plus for 12 h at 55 °C. Longitudinal Sects. (5–7 μm thick) were cut from the hypocotyls using a Spencer 820® rotary microtome and stained with Safranin O and Alcian blue staining solution to highlight the primary and secondary walls, respectively. Some sections were stained with Amido Black staining solution 2× and toluidine blue O for additional visualization. All samples were examined using a photonic microscope.

#### Rooting and plant acclimatization

Once shoots reached 3 cm in length, they were separated from the explants and planted in WPM with IBA and NAA (two concentrations). Control experiments were also set up without growth regulators. In the fourth week, the number of rooted plants and the average number of roots per plant were estimated. Plants 6 cm in height with roots were removed from the medium, washed with abundant water, and transferred to individual pots (9 × 10 cm) in a mixture of peat moss and sand (3:1) that had previously been sterilized. Pots were transferred to a greenhouse and covered with perforated plastic glass for 20 d. Every 4 d, the substrate was moistened, and every week it was fertilized with WPM basal salts, Murashige and Skoog [14] basal salts, or just water. On the 30th day, the survival percentage was estimated. On the 60th day, plant height, the number of leaves, shoots, roots, and survival percentages were assessed.

#### DNA extraction and RAPD amplification

The clonal fidelity of in vitro–raised plants was tested using RAPD markers. DNA was extracted using the cetyltrimethylammonium bromide (CTAB) method [15] and the DNA quality and quantity was verified on agarose gel using a Nanodrop 2000 spectrophotometer. Fifteen RAPD primers were used under the conditions reported by Gajera et al. [16]. Each experiment was repeated three times, and reproducible DNA bands were considered for data analysis. The presence (1) or absence (0) of fragments was manually scored in a binary data matrix. Amplified fragments were analyzed as alleles, assuming Hardy–Weinberg equilibrium and loci segregation in a dominant Mendelian fashion. We used Jaccard's coefficient of similarity [Jij = a/(a + b + c)] to compute the binary datasets [17].

#### Statistical analysis

Data normality was investigated [18] and differences between pairs of groups were evaluated using the T-test. Differences between more than two groups were evaluated [19] using analysis of variance (ANOVA) for parametric data and the Kruskal–Wallis test [19] for non-parametric distributions followed by the pairwise Wilcoxon rank-sum [20] or the Tukey HSD test, respectively, when at least one different group was found (α < 0.05). All statistical analyses were performed using R 3.4.2 software [21].

### Results and discussion

#### Culture initiation and histological analysis

**Fig. 1:**
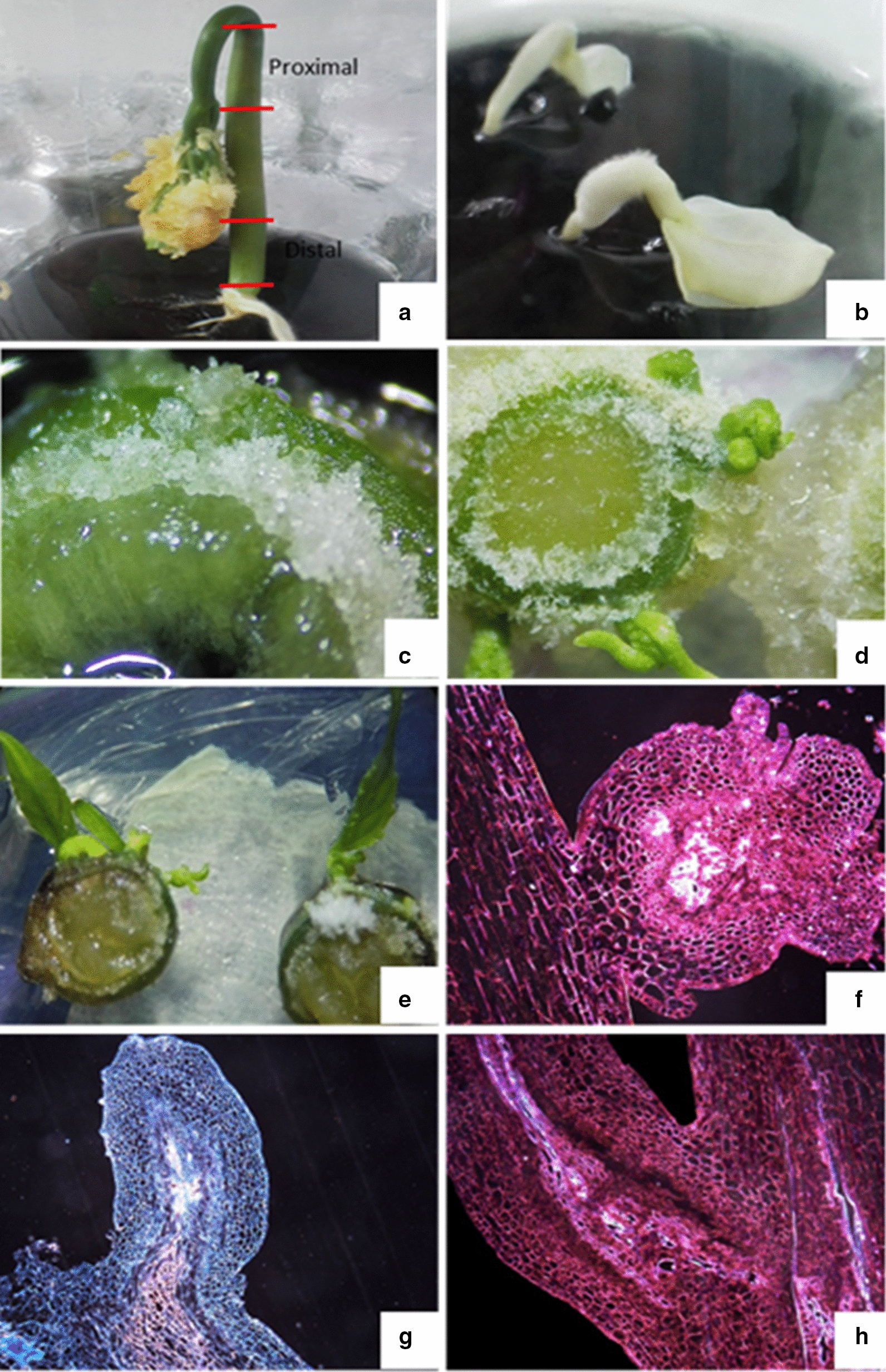
**a** Hypocotyls of 2-week-old *Plukenetia volubilis* plantlets obtained through in vitro germination. **b** Rescue of embryos. **c.** Callus formation on hypocotyls at 8 d of culture. **d** Proliferation of adventitious shoots from hypocotyls **e** Development of adventitious shoots. **f** Development of nodular structure on the hypocotyl surface. Scale bar = 200 mm. **g** Connection between the hypocotyl and nodular structure through vascular bundles. Scale bar = 200 mm. **h** Periclinal and anticlinal divisions. Scale bar = 200 mm

Due to high seed contamination (35.6%) and a low germination rate (34.4%) (Fig. 1a), mature zygotic embryo rescue was necessary (Fig. 1b), which led to an increase in the germination rate to 71.7%. Hypocotyl segments selected as explants from 3-week-old plantlets (Fig. 1a) developed calluses after 8 d of culture (Fig. 1c). After 20 d, adventitious shoots appeared in all treatment groups, including the control (Fig. 1d–e). All segments treated with growth regulators developed calluses, and histological analysis indicated a connection between maternal tissue and induced shoots not from the callus, which is characteristic of the organogenic process and demonstrates organogenic growth (Fig. 1f–h). The central part of the nodular structure was mainly surrounded by parenchyma, cambium, and tracheal elements (Fig. 1h). The connection zone between the hypocotyl and the nodular structure presented small cells with huge periclinal and anticlinal divisions, massive cores, and dense cytoplasm (Fig. 1f–h); meanwhile, the epidermal and sub-epidermal layers comprised cells that were divided and more intensely stained. The formation of meristematic areas from differentiated cells occurred due to the proximity of differentiated cells to existent meristematic tissues, where growth regulators were assumed to be highly concentrated [22].

#### Shoot induction and regeneration of plantlets

Shoot induction from hypocotyl segments depended on the position (proximal/distal) relative to the cotyledonary node (Table 1). A higher number of induced shoots were observed closer to the cotyledonary node, as previously reported in *Jatropha curcas* [5] and *Euonymus japonicus* [23]. Shoot number gradually and significantly decreased (p ≤ 0.05) with distance from the cotyledonary node, possibly due to the lower level of endogenous growth regulators further from the node.

Although severe adventitious shoot formation was observed at all concentrations of growth regulators, a more significant rate (5.1 shoots per hypocotyl proximal section) was observed in medium containing 1 mg/L TDZ and 0.5 mg/L zeatin and supplemented with G + A + A. Meanwhile, the lowest adventitious shoot formation rate was obtained for the control medium supplemented with CuSO_4_ and G + A + A. In the hypocotyl distal section, the average number of shoots per explant was comparable at all growth regulator concentrations, with no significant differences between treatments (p ≤ 0.05). Cytokinins, particularly TDZ, are hormones that directly or indirectly stimulate organogenesis, promoting de novo shoot regeneration in herbaceous [24] and woody plants [25]. In species from the Euphorbiaceae family, such as *J. curcas* and *Ricinus communis*, TDZ has been reported to induce the formation of adventitious shoots from hypocotyls [5, 26].

#### Rooting and acclimatization

The number of rooted plants was highest on the 45th day, with the highest percentage of rooted plants (91.1%) in 1 mg/L IBA and the lowest (48.9%) in the control medium (p ≤ 0.05) (Table S1). Root formation occurred in all treatment groups, probably due to the endogenous concentration of auxins or activated charcoal supplements, which produce ideal conditions for root development in plants [27]. IBA has been extensively used to induce rooting in different species under in vitro conditions, with optimum results at low concentrations [28]. In Euphorbiaceae, IBA has been reported to induce rooting at low concentrations in *J. curcas* (0.2 mg/L IBA) [29] and *R. communis* (1 mg/L IBA) [26], although other species require high IBA concentrations (3.0 mg/L) [30].

Plants with well-developed roots were transplanted to a greenhouse in which the humidity and luminous intensity were semi-controlled. There, sacha inchi plants showed excellent acclimatization, with a survival percentage of > 80%. After 2 months, plants treated with WPM nutrients reached 7.96 cm in height (Table 2). The peat moss used as substrate probably played a considerable role in minimizing hydric stress in hardened plants. At the end of the process, the regenerated plants showed no signs of morphological abnormalities.

**Table 2:** Survival percentage, average height, and number of leaves, roots, and shoots of *Plukenetia volubilis* plants germinated in vitro at 60 d of hardening

Media treatments	Survival (%)	Average height (cm)	# Average leaves	# Average roots
WPM	86.67^a^	7.96^a^	4.4^a^	2.38^a^
MS	82.22^a^	6.69^b^	3.29^b^	1.2^b^
Control	84.44^a^	4.38^c^	2.18^c^	1.02^b^

Different letters indicate significant differences (p ≤ 0.05)

#### RAPD analysis

RAPD analysis has been widely used to characterize somaclonal variation in plantlets in vitro because it is affordable and useful for detecting variations in vegetable crops [31]. In the present study, the RAPD markers OPD-08 and OPA-13 allowed the amplification of 17 fragments from all genotypes analyzed, with sizes between 250 and 4000 bp. Only three fragments, obtained with the primer OPA-13, were polymorphic; these fragments exhibited insignificant genetic variability between mother and daughter plants, confirming the genetic stability of the sacha inchi plantlets generated through direct in vitro organogenesis from hypocotyls. Similar results were previously obtained in *Musa acuminata* [32] and *Solanum melongena* L.[33], which were regenerated from hypocotyls with no evidence of polymorphic bands in RAPD analysis. According to Jaccard's coefficient of similarity, the plants regenerated via organogenesis in the present study had 89% genetic similarity to mother hypocotyls (Table S2), indicating that the micropropagation procedure reliably maintained the genetic stability of plant material.

## Limitations

Further work needs to be done to establish genetic stability using additional molecular markers (e.g., microsatellites) or, ideally, flow cytometry. This research did not examine in detail the role played by parameters such as age, cultivar genotype, and the concentrations of plant growth regulators on regeneration efficiency.

## Supplementary information

**Additional file 1: Table S1. ** Effect of auxins on the rooting of adventitious shoots in *Plukenetia volubilis*

**Additional file 2: Table S2. **Similarity coefficients among mother plants and micropropagated hypocotyls of *Plukenetia volubilis* based on RAPD markers.

## Data Availability

The datasets used and/or analyzed during the current study are available from the corresponding author on reasonable request.

## Abbreviations

2ip: 6-γ, γ-Dimethylallylamino purine
ANLA: National Authority for Environmental licenses
BAP: 6-Benzylaminopurine
CTAB: Cetyltrimethylammonium bromide
CuSO_4_: Cupric sulfate pentahydrate
G + A + A: l-Glutamine, adenine hemisulfate, and L-arginine
IBA: Indole-3-butyric acid
NAA: 1-Naphthylacetic acid
MS: Murashige and Skoog
RAPD: Random amplification of polymorphic DNA
TDZ: Thidiazuron
WPM: Woody plant medium
