# Supplementary material for: Efficient direct shoot organogenesis and genetic stability in micropropagated sacha inchi (Plukenetia volubilis L.)
Source: BMC Res Notes. 2020 Sep 3;13:414. doi: 10.1186/s13104-020-05257-1 (PMC7650214; doi:10.1186/s13104-020-05257-1)
Supplement: Supplementary file 1 — Additional file 1: Table S1. Effect of auxins on the rooting of adventitious shoots in Plukenetia volubilis [file 13104_2020_5257_MOESM1_ESM.docx]

**Table S1.** Effect of auxins on the rooting of adventitious shoots in *P. volubilis*

| **Hormonal Treatments** | **Concentration (mg/L)** | **Rooting (%)** | **Average of roots per explant** |
| --- | --- | --- | --- |
| **IBA** | 0.5 | 80 ^b^ | 1,47 ^b^ |
|  | 1 | 91,11^b^ | 2,02 ^c^ |
| **NAA** | 0.5 | 71,11 ^ab^ | 1,31 ^ab^ |
|  | 1 | 73,33^ab^ | 1,56^bc^ |
| **Control** | - | 48,89 ^a^ | 0,78 ^a^ |

Different letters indicate significant differences (p ≤ 0.05).
