# Supplementary material for: Efficient direct shoot organogenesis and genetic stability in micropropagated sacha inchi (Plukenetia volubilis L.)
Source: BMC Res Notes. 2020 Sep 3;13:414. doi: 10.1186/s13104-020-05257-1 (PMC7650214; doi:10.1186/s13104-020-05257-1)
Supplement: Supplementary file 2 — Additional file 2: Table S2. Similarity coefficients among mother plants and micropropagated hypocotyls of Plukenetia volubilis based on RAPD markers. [file 13104_2020_5257_MOESM2_ESM.docx]

**Table S2.** Similarity coefficients among mother plant and micropropagated hypocotyls of *P. volubilis* based on RAPD markers.

| **Mother** | **OPD-08 S1** | **OPD-08 S2** | **OPA-13 S1** | **OPA-13 S2** | **OPD-08 & OPA-13 S1** | **OPD-08 & OPA-13 S2** | **Average** |
| --- | --- | --- | --- | --- | --- | --- | --- |
| H35 | 1 | 1 | 1 | 1 | 0.67 | 0.67 | 0.89 |
| H36 | 1 | 1 | 1 | 1 | 0.71 | 0.71 | 0.90 |
| H42 | 1 | 1 | 1 | 0.67 | 0.83 | 0.83 | 0.89 |
| H43 | 1 | 1 | 1 | 1 | 0.86 | 0.86 | 0.95 |
| H44 | 1 | 1 | 1 | 0.8 | 1 | 1 | 0.97 |
| H47 | 1 | 1 | 1 | 1 | 0.86 | 1 | 0.98 |
| H48 | 1 | 1 | 1 | 1 | 1 | 1 | 1 |
| Average | 1 | 1 | 1 | 0.92 | 0.85 | 0.87 | 0.94 |
